# Cyclin-dependent kinase-mediated phosphorylation and the negative regulatory domain of transcription factor B-Myb modulate its DNA binding

**DOI:** 10.1016/j.jbc.2022.102319

**Published:** 2022-08-01

**Authors:** Tilini U. Wijeratne, Keelan Z. Guiley, Hsiau-Wei Lee, Gerd A. Müller, Seth M. Rubin

**Affiliations:** Department of Chemistry and Biochemistry, University of California, Santa Cruz, California, USA

**Keywords:** cell cycle, gene regulation, Cdk, intrinsically disordered protein, autoregulation, CHR genes, CHR, cell-cycle genes homology region, CycA, CyclinA, DBD, DNA-binding domain, FP, florescence polarization, IDR, intrinsically disordered region, ITC, isothermal titration calorimetry, MBD, MuvB-binding domain, MBS, Myb-binding site, MMB, Myb-MuvB, NRD, negative regulatory domain, TAD, transactivation domain, TF, transcription factor

## Abstract

B-Myb is a highly conserved member of the vertebrate Myb family of transcription factors that plays a critical role in cell-cycle progression and proliferation. Myb proteins activate Myb-dependent promoters by interacting specifically with Myb-binding site (MBS) sequences using their DNA-binding domain (DBD). Transactivation of MBS promoters by B-Myb is repressed by its negative regulatory domain (NRD), and phosphorylation of the NRD by Cdk2-CyclinA relieves the repression to activate B-Myb–dependent promoters. However, the structural mechanisms underlying autoinhibition and activation of B-Myb–mediated transcription have been poorly characterized. Here, we determined that a region in the B-Myb NRD (residues 510–600) directly associates with the DBD and inhibits binding of the DBD to the MBS DNA sequence. We demonstrate using biophysical assays that phosphorylation of the NRD at T515, T518, and T520 is sufficient to disrupt the interaction between the NRD and the DBD, which results in increased affinity for MBS DNA and increased B-Myb–dependent promoter activation in cell assays. Our biochemical characterization of B-Myb autoregulation and the activating effects of phosphorylation provide insight into how B-Myb functions as a site-specific transcription factor.

The Myb family of transcription factors (TFs) are present in a range of species from slime mold to higher eukaryotes and have high conservation in their DNA-binding domain (DBD) (1, 2, 3, 4). TFs with evolutionary conserved DBDs recognize a common DNA sequence; however, they often diverge in their distinct functions through different intra- and inter-molecular interactions (5). Vertebrate Myb family members A-Myb, B-Myb, and c-Myb share more than 70% amino sequence homology in their DBDs, which recognize the Myb-binding site (MBS) DNA sequence (C/TAACNG) (4, 6, 7, 8). All the Myb family members regulate transcription of genes important for cell differentiation and proliferation, but they differ in their tissue-specific expression. A-Myb (encoded by *MYBL1*) is mainly expressed in cells of the developing central nervous system, sperm cells, and breast tissue, while c-Myb (*MYB*) is expressed specifically in immature hematopoietic stem cells (9, 10). In contrast, B-Myb (*MYBL2*), which is the most ancient of the paralogs, is ubiquitously expressed in all proliferating cells (11). The prominent role of B-Myb in both differentiating and proliferating cells is reflected by its deregulation in several cancers. Overexpression of the *MYBL2* gene is considered a biomarker for poor prognosis in osteosarcoma, breast cancer, esophageal cancer, and multiple myeloma (12, 13, 14, 15). Therefore, in recent years, B-Myb has become an attractive target to understand mechanistic details of oncogenic TFs for cancer therapeutics.

The B-Myb domain architecture is similar to A-Myb and c-Myb. B-Myb contains a DBD, a negative regulatory domain (NRD) toward the C-terminus, and a transactivation domain (TAD) (Fig. 1*A*). In all three Myb proteins, C-terminal protein truncations that remove the NRD trigger activation of Myb-dependent reporter promoters (16, 17, 18). Recurrent chromosomal translocations involving the genes *MYB* and *MYBL1* produce NRD truncated versions of the respective proteins c-Myb and A-Myb that are sufficient to induce leukemias in mice (19, 20). These truncated proteins more resemble the viral oncoprotein v-Myb, which shares the same DBD as all the Myb proteins and has a TAD but lacks a potent C-terminal NRD (21). In contrast, C-terminal truncations of B-Myb are not reported to have oncogenic properties. However, the B-Myb C-terminus contains the MuvB-binding domain (MBD), which binds the MuvB complex to assemble Myb-MuvB (MMB) (22, 23, 24). The MMB complex activates cell-cycle–dependent genes that contain a CHR sequence (cell-cycle genes homology region) in their promoter in a manner that is both B-Myb and MuvB dependent (24). Thus, B-Myb functions as a site-specific TF that activates MBS genes and as a coactivator of CHR genes when present in the MMB complex. This latter function is unique among Myb family members.

**Figure 1:**
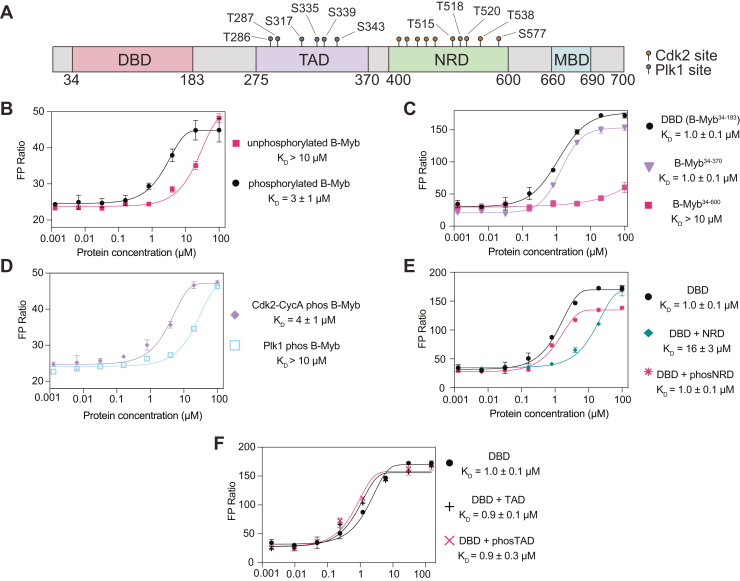
**Phosphorylation by Cdk2-CycA enhances B-Myb binding to MBS DNA.***A*, a schematic presenting the boundaries of the DBD (DNA-binding domain), TAD (transactivation domain), NRD (negative regulatory domain), and MBD (MuvB-binding domain). Previously identified Cdk2 and Plk1 sites are indicated. *B*, fluorescence polarization (FP) assay of B-Myb binding to a TAMRA-labeled MBS probe. The measurements compare unphosphorylated B-Myb to B-Myb that was sequentially phosphorylated by Cdk2-CyclinA and Plk1. *C*, same FP assay used to measure binding affinities of C-terminal truncations of B-Myb to the MBS probe. *D*, same FP assay used to measure probe affinity of B-Myb phosphorylated only by Cdk2-CyclinA or Plk1. *E*, FP assay of DBD binding to MBS probe as in panel (*C*) but also performed by using DBD incubated with 30 μM NRD or phosNRD. *F*, as in panel (*E*) but using DBD incubated with 30 μM TAD or phosTAD. MBS, Myb-binding site.

The mechanisms by which the NRD affects the transactivation potential of B-Myb are also not yet fully understood. Several studies show that autoinhibition of B-Myb by the NRD is relieved when B-Myb is phosphorylated by the cell-cycle regulatory kinase Cdk2-CyclinA (Cdk2-CycA), which results in the activation of MBS-dependent promoters (17, 25, 26). The NRD of Myb proteins contains several highly conserved Cdk consensus sites (S/TP) (Figs. 1*A* and S1), including a TPTPFK motif (amino acids 518–523 in B-Myb). This region is a direct target of Cdk-mediated phosphorylation in cell-based studies and shows a positive correlation with activation (27, 28). Cdk-dependent phosphorylation of B-Myb also primes for binding of Polo-like kinase 1 (Plk1), and subsequent phosphorylation by Plk1 in the TAD also promotes B-Myb activity (29). Although evidence suggests that NRD phosphorylation releases an inhibited state of B-Myb to significantly transactivate MBS-dependent promoter reporters, two studies did not find that Cdk2-CycA alters B-Myb interactions with DNA (30, 31). However, evidence of enhanced DNA binding upon truncation of the NRD in A-Myb and c-Myb has been reported (16, 18). A recent study showed that the B-Myb DBD undergoes an intramolecular interaction with the NRD. Cdk-mediated phosphorylation at a specific site (S577) disrupted the interaction; however, it was not conclusive if the NRD–DBD interaction affected the ability of B-Myb to bind DNA and if phosphorylation regulated the NRD-DBD association (31). B-Myb phosphorylation is not only required for MBS-dependent transactivation, but it is also important for G2/M cell cycle–dependent gene activation. B-Myb is extensively phosphorylated by Cdk2-CycA during S phase of the cell cycle coinciding with its peak in expression (24, 29). On the other hand, other studies conclude that extensive phosphorylation of B-Myb is important for its ubiquitination and proteasomal mediated degradation (32). Thus, despite these various studies, it remains unclear how phosphorylation of B-Myb overcomes negative regulation by the NRD to activate B-Myb. In part, disparate models have arisen because of the challenges of interpreting cell-based assays to draw conclusions about direct molecular interactions and the effects of phosphorylation on specific interactions.

Here, we present a study of B-Myb autoregulation that focuses on biophysical assays using purified proteins. We used recombinant expression, which provided us with a minimal system to control phosphorylation, and we quantified interactions by measuring dissociation constants. We found that the B-Myb NRD binds the DBD with a low micromolar affinity, and the interaction is sufficient to inhibit B-Myb binding to MBS DNA. We identified amino acids that are critical for NRD-DBD association and observed that Cdk2-CycA–mediated phosphorylation of T515, T518, and T520 disrupts the NRD–DBD interaction to enhance binding to an MBS DNA sequence probe. We also show that specific mutations that disrupt the NRD–DBD interaction increase B-Myb–dependent activation of an MBS luciferase reporter. Our findings reveal a structural mechanism for B-Myb autoregulation of site-specific gene activation and how repression is relieved by Cdk phosphorylation.

## Results

### Phosphorylated B-Myb binds to DNA tighter than unphosphorylated B-Myb

In order to determine the effects of B-Myb phosphorylation on its association with DNA, we used fluorescence polarization (FP) assays to measure binding affinities using recombinant, purified proteins. Considering that both Cdk2-CycA and Plk1 sites are present throughout the NRD and TAD regions, respectively (Figs. 1*A* and S1) (27, 29, 30), we sequentially phosphorylated purified, full-length B-Myb. We phosphorylated first with Cdk2-CycA and then with Plk1, and we verified phosphorylation with a mobility shift on a PhosTag gel (Fig. S2, *A* and *B*). We then assayed DNA binding of phosphorylated and unphosphorylated protein to a fluorescently labeled DNA probe containing the MBS sequence. The resulting single-site protein-DNA binding curve for the phosphorylated B-Myb showed a dissociation constant K_D_ = 3 ± 1 μM (Fig. 1*B*). The unphosphorylated B-Myb showed weak (K_d_ > 10 μM), potentially nonspecific, binding to the probe.

### Phosphorylation of the NRD regulates DBD binding to DNA

We found that the affinity of phosphorylated B-Myb for the MBS probe was similar to the affinity of the DBD alone (B-Myb^34–183^, K_D_ = 1.0 ± 0.1 μM) and to the affinity of a construct in which the NRD was deleted (B-Myb^34–370^, K_D_ = 1.0 ± 0.1 μM) (Fig. 1*C*). In contrast, a C-terminal truncation of the MBD that leaves the NRD intact (B-Myb^34–600^) bound with similar weak affinity as unphosphorylated full-length B-Myb (Fig. 1*C*). The observation that binding of the construct containing the DBD-TAD-NRD domains is greatly reduced compared to both the DBD and DBD-TAD only constructs demonstrates that the NRD inhibits DBD binding to the MBS probe. These results and the fact that there are no Cdk sites in the DBD support a model in which the DBD–DNA interaction is inhibited by the NRD in the context of unphosphorylated B-Myb and that this inhibition is relieved upon Cdk2-CycA–mediated phosphorylation of the NRD. To test this model, we phosphorylated B-Myb with Cdk2-CycA only and found a similar affinity as phosphorylating with both kinases. In contrast, phosphorylation with only Plk1 had no effect on the binding compared to unphosphorylated B-Myb (Figs. 1*D* and S2).

To further test the role of the NRD in inhibiting DBD-DNA binding and the role of NRD phosphorylation, we performed FP assays, titrating DBD into DNA in the presence of unphosphorylated and Cdk2-phosphorylated NRD^440–600^ (verified by mass spectrometry, Fig. S2*C*). As shown in Figure 1*E*, when added *in trans*, 30 μM unphosphorylated NRD reduced DBD binding to the MBS probe (K_D_ = 16 ± 3 μM). In contrast, when 30 μM phosphorylated NRD was added *in trans*, there was little effect of the NRD on the affinity of the DBD for the MBS probe (K_D_ = 1.0 ± 0.1 μM). The addition of Plk1-phosphorylated TAD *in trans* also did not influence DBD binding to the MBS probe (Figs. 1*F* and S2*D*). These data further support the model that Cdk phosphorylation of NRD^440–600^ and not Plk1 phosphorylation of sites in the TAD increases the affinity of DNA binding through release of autoinhibition.

### Direct association of the NRD with the DBD

**Figure 2:**
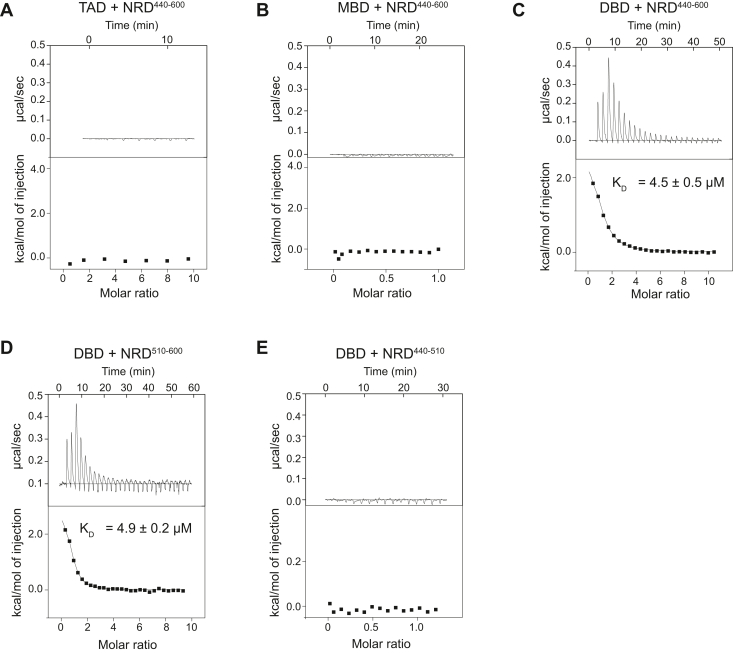
**B-Myb NRD**^**510–600**^**directly binds DBD.***A*, isothermal titration calorimetry (ITC) binding measurement between NRD^440–600^ and TAD. *B*, ITC binding measurement between NRD^440–600^ and MBD. *C*, ITC-binding measurement between NRD^440–600^ and DBD. *D*, ITC-binding measurement between NRD^510–600^ and DBD. *E*, ITC-binding measurement of NRD^440–510^ and DBD. DBD, DNA-binding domain; MBD, MuvB-binding domain; TAD, transactivation domain.

We next probed the presence of intramolecular interactions within B-Myb that may drive the observed autoinhibition of DNA binding. We mixed separately purified domains and detected interdomain association by isothermal titration calorimetry (ITC). We observed no detectable binding of the TAD or MBD to an NRD construct that contains amino acids 440 to 600 (NRD^440–600^, Fig. 2, *A* and *B*). In contrast, we detected association of the DBD and NRD^440–600^ and measured an affinity of K_D_ = 4.5 ± 0.5 μM (Fig. 2*C*). To map a more minimal NRD, we used sequence conservation to divide the NRD into two halves (Fig. S1). We observed that the C-terminal half (amino acids 510–600, B-Myb^510–-600^) binds the DBD with a similar affinity of K_D_ = 4.9 ± 0.2 μM (Fig. 2*D*), and we did not observe association of the N-terminal half (B-Myb^440–510^, Fig. 2*E*).

### NMR spectroscopy mapping of the amino acids mediating the B-Myb DBD–NRD interaction

**Figure 3:**
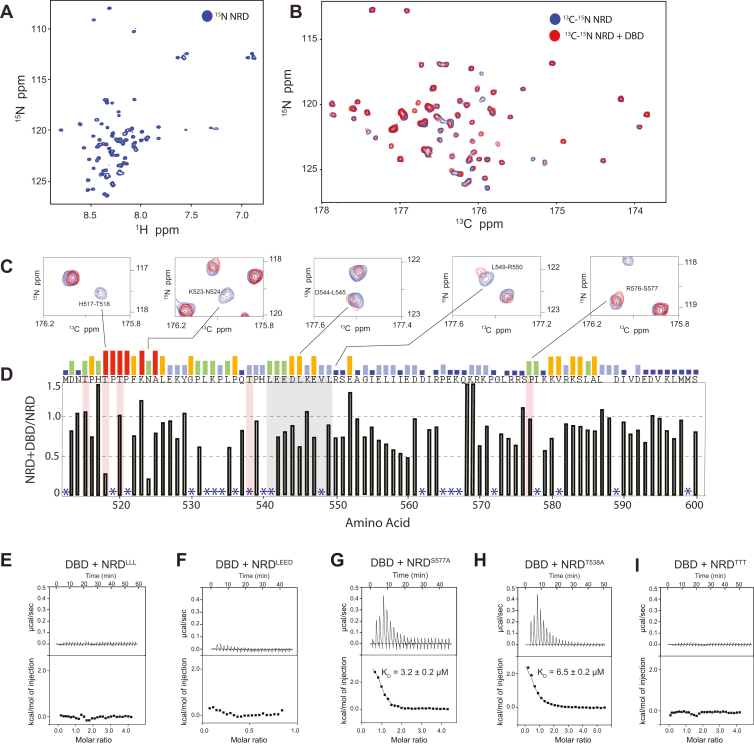
**NMR spectroscopy maps potential NRD residues that interact with DBD.***A*, ^1^H-^15^N HSQC spectrum of labeled NRD^510–600^ at 300 μM. *B*, ^13^C-^15^N CON spectra of labeled NRD^510–600^ at 300 μM alone (*blue*) and with 600 μM unlabeled DBD (*red*). *C*, close-up views of exemplary assigned peaks in the ^13^C-^15^N CON spectra showing significant peak broadening. *D*, relative intensity of each amino acid plotted as the ratio of the intensity of NRD alone to the intensity of NRD + DBD. *Asterisks* mark amino acids that could not be assigned. Residues marked in *red* are Cdk consensus phosphorylation sites. Relative sequence conservation through Myb family members is displayed by the height of the bars above the primary sequence at the top of the graph. The sequence highlighted in *gray* corresponds to a region of high conservation and is the focus of our following mutagenesis studies. See Fig. S1 for the full NRD sequence alignment and Fig. S3 or analysis of chemical shift perturbations. *E*–*I*, ITC-binding measurements of DBD to mutant NRD^510–600^ constructs. LLL refers to L541, L545, and L549, LEED refers to L541, E542, E543, D544, and TTT refers to T515A, T518A, T520A. DBD, DNA-binding domain; ITC, isothermal titration calorimetry; NRD, negative regulatory domain.

We next used NMR to further probe the NRD^510–600^–DBD interaction. The minimal chemical shift dispersion of the ^1^H-^15^N HSQC spectrum of ^15^N-labeled B-Myb^510–600^ suggests that the fragment is structurally disordered (Fig. 3*A*). We therefore generated a ^13^C-^15^N double-labeled sample and proceeded with ^13^C-^15^N CON spectroscopy, which is well suited for studying interactions of intrinsically disordered proteins (33). The two-dimensional CON spectrum contains crosspeaks at the chemical shifts of the backbone carbonyl carbon and amide nitrogen and is typically better dispersed than the ^1^H-^15^N HSQC spectrum. To observe the NRD-DBD association, we added isotopically unlabeled DBD to ^13^C-^15^N-labeled NRD and monitored the chemical shift perturbations in a two-dimensional CON spectrum. A number of cross-peaks showed changes in both intensity and position, which is consistent with the binding we observed by ITC (Fig. 3*B*). The most pronounced perturbations appear as loss of intensity, which reflects peak broadening from either intermediate exchange or from the NRD^510–600^ forming a larger molecular weight complex when bound by the unlabeled DBD.

We assigned the CON spectrum using standard backbone correlation experiments, and these assignments enabled identification of amino acid sequences in the NRD^510–600^ that are potentially critical for DBD binding (Fig. 3, *C* and *D*). Plots of peak intensity loss (Fig. 3*D*) and chemical shift change (Fig. S3) upon addition of DBD to the NRD^510–600^ sample show that perturbations occurred at regions throughout NRD^510–600^. We were particularly interested in the perturbations that clustered around residues 514 to 526 and 542 to 547 (Figs. 3*D* and S1). These clusters of residues show broadening, and the sequences are relatively well conserved. In addition, the sequence between 538 and 565 has helical propensity, and analysis suggests a hydrophobic surface containing several leucines that are conserved in B-Myb orthologs (Fig. S4). We surmised that if formed upon binding, such a helix would be a good candidate for facilitating interdomain interactions. To determine whether these regions are important for NRD-DBD association, we made two sets of alanine mutations in the most conserved residues found in these regions; we mutated together L541, E542, E543, D544 (NRD^LEED^) and together L541, L545, and L549 (NRD^LLL^). We expressed and purified the mutant NRD^510–600^ constructs and tested binding to DBD using ITC. We found that these NRD mutant domains do not bind to DBD (Fig. 3, *E* and *F*). We note that mutations in the NRD do not markedly perturb the overall NMR spectrum, suggesting that the ensemble of disordered NRD conformations remains intact (Fig. S5). Together, these mutagenesis and NMR data support the conclusion that residues within 540 to 550 make critical contacts with the DBD.

There are five consensus Cdk2-CycA phosphorylation sites in NRD^510–600^ (T515, T518, T520, T538, and S577), and all of these phosphosites except T538 have been validated by two-dimensional tryptic peptide mapping and point mutagenesis (17, 30, 34, 35). In our NMR spectra, we were unable to assign all the phosphorylation sites due to repetitive amino acid sequences, but we successfully assigned S577 and T518 (Fig. 3*D*). We observed a substantial change in intensity for the peak corresponding to T518 and for peaks corresponding to nearby residues (N514, T515, H517, T518) when DBD was added (Fig. 3, *C* and *D*). In contrast, we observed minimal perturbations for the S577 peak and peaks corresponding to the residues around S577, which were reported to disrupt the NRD–DBD interaction when deleted (29). To test the role of these phosphorylation sites in the NRD–DBD interaction, we created three C-terminal NRD^510–600^ fragments with different phosphosites mutated to alanine (NRD^S577A^, NRD^T538A^, and NRD^TTT^, which contains T515A, T518A, T520A). We used these mutated and not phosphorylated NRDs in ITC experiments to detect binding affinities with DBD. We found that both NRD^S577A^ and NRD^T538A^ bound to DBD with K_D_ = 3.2 ± 0.2 μM and K_D_ = 6.5 ± 0.2 μM, respectively (Fig. 3, *G* and *H*). However, NRD^TTT^ showed no detectable binding, indicating that these threonines, when unphosphorylated, are important to interact with DBD (Fig. 3*I*).

### Phosphorylation of Cdk consensus sites in the conserved region of the NRD modulates the NRD–DBD interaction to regulate DNA binding

**Figure 4:**
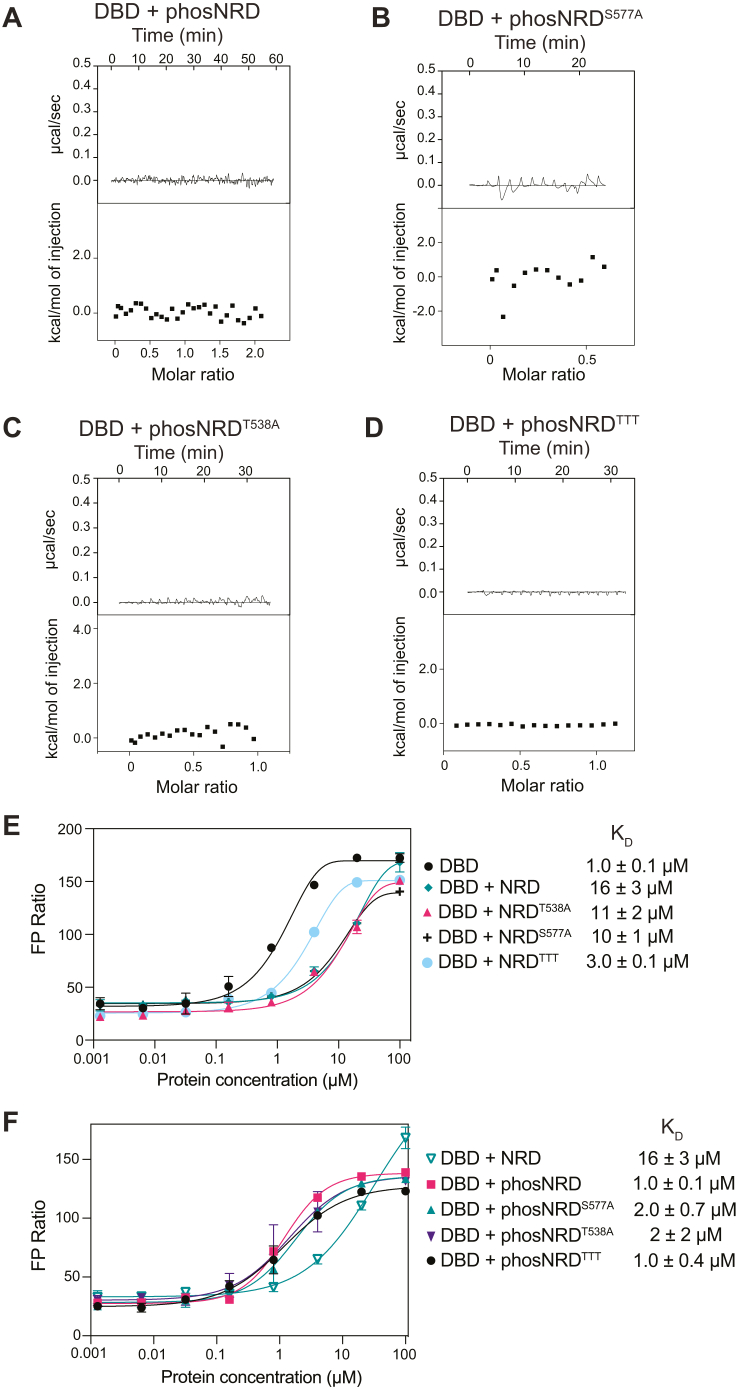
**Phosphorylated NRD does not interact with DBD allowing DBD to interact with MBS DNA.***A*–*D*, ITC measurements of DBD binding to the indicated NRD construct after phosphorylation by Cdk2-CyclinA. Phosphorylation of NRD was confirmed through electrospray mass spectrometry shown in Fig. S1. *E* and *F*, FP assay measurements of DBD binding to the TAMRA-labeled MBS probe incubated with the indicated NRD construct at 30 μM. DBD, DNA-binding domain; FP, florescence polarization; ITC, isothermal titration calorimetry; MBS, Myb-binding site; NRD, negative regulatory domain.

We next tested to what extent phosphorylation of Cdk sites in NRD^510–600^ influences NRD binding to the DBD and the inhibition of DBD-DNA binding. We phosphorylated purified NRD^510–600^ constructs with Cdk2-CycA and tested NRD-DBD affinities using ITC (Fig. 4). We verified phosphorylation of the WT NRD^510–600^ (B-Myb 510–600) on five sites with electrospray mass spectrometry (Fig. S2*E*). We observed no detectable binding toward DBD when WT NRD was phosphorylated (Fig. 4*A*). Similarly, we observed that phosphorylated NRD^S577A^ and NRD^T538A^ did not bind to DBD, indicating that phosphorylation of those specific sites is not required and that phosphorylation of T515A, T518A, T520A is sufficient to disrupt the interaction (Fig. 4, *B* and *C*). Phosphorylation of NRD^TTT^ also resulted in no binding to DBD (Fig. 4*D*), although we had already established that these threonine residues are critical for the association when the protein is unphosphorylated (Fig. 3*I*).

We performed the FP-binding assay with fluorescently labeled MBS probe and added the various WT and phosphorylation-site mutant NRD^510–600^ constructs *in trans* (Fig. 4, *E* and *F*). As previously shown in Figure 1*F*, the DBD alone binds to the MBS probe with K_D_ of 1.1 ± 0.1 μM and when NRD is added *in trans* to DBD, the affinity decreases to a K_D_ of 16 ± 3 μM. We found that, when unphosphorylated, the mutants NRD^S577A^ and NRD^T538A^ still inhibited DBD binding to the MBS probe. When NRD^TTT^ was added *in trans*, DBD-binding affinity to the MBS probe was more weakly inhibited, consistent with our observation that T515, T518, and T520 are important for the interaction between the NRD and DBD that inhibits DBD binding to DNA (Fig. 4*E*). Phosphorylation of the NRD^S577A^ and NRD^T538A^ constructs with Cdk2-CycA abrogated their inhibitory effect on DNA binding, but phosphorylation of NRD^TTT^ resulted in DNA-binding inhibition similar to the unphosphorylated mutant (Fig. 4*F*). Together, these FP and ITC results are consistent with a model in which phosphorylation of T515, T518, T520 modulates the association of the NRD with the DBD in a manner that can regulate DNA-binding affinity. Considering that neither mutation of the threonine phosphorylation sites nor phosphorylation of the WT NRD results in widespread changes to NMR spectra that would suggest overall structural changes (Fig. S5), we favor the interpretation that these threonine residues make specific contacts with the DBD that are broken upon mutation or phosphorylation.

### Disruption of the NRD–DBD interaction increases the transactivation potential of B-Myb

**Figure 5:**
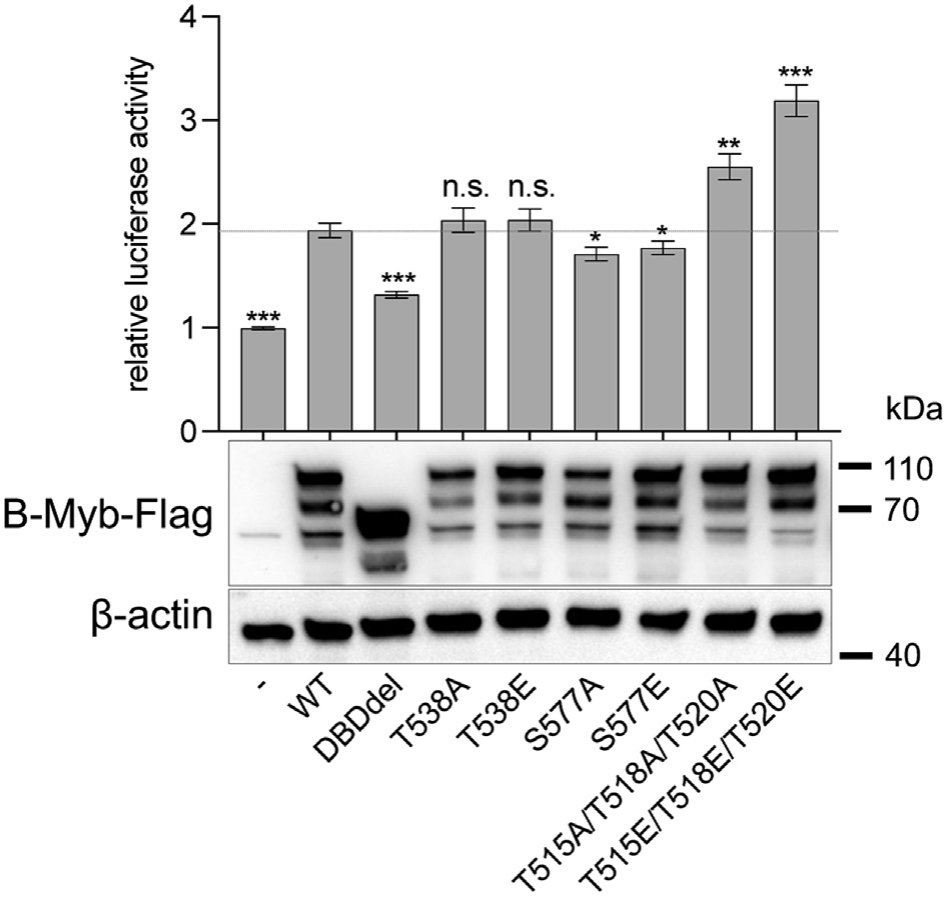
**Disruption of a critical NRD-DBD interface hyperactivates B-Myb.** HCT116 cells were transfected with a luciferase reporter plasmid containing three Myb-binding sites (MBSs) upstream of a minimal promoter together with plasmids expressing Flag-tagged wild-type (WT) B-Myb or the indicated mutants. DBDdel is mutant with entire DNA-binding domain deleted (amino acids 12-182). Mean values ± SD of four biological replicates are given, and significances were calculated by the Students paired *t* test (∗*p* ≤ 0.05, ∗∗*p* ≤ 0.01, ∗∗∗*p* ≤ 0.001 compared with WT B-Myb). Expression levels of B-MYB variants in the luciferase assay samples were analyzed by SDS-PAGE/Western blot. DBD, DNA-binding domain; NRD, negative regulatory domain.

To probe the functional significance of the NRD–DBD interaction in B-Myb–mediated transcriptional activation, we performed luciferase reporter assays in HCT116 cells (Fig. 5). Plasmids encoding WT and mutant B-Myb were transfected along with the pGL4.10 luciferase reporter plasmid containing an artificial promoter with three MBS consensus sequences. Such constructs have already been utilized to detect B-Myb–dependent gene activation (36, 37). As previously described, we observe a positive effect of B-Myb on the activity of the MBS promoter and a significant decrease of activation when the DBD is deleted. We tested mutation of phosphorylation sites in the NRD that we found to be important for NRD-DBD association in the NMR and ITC assays (Figs. 3 and 4). We found that B-Myb with mutation of the three NRD Cdk site threonines (T515/T518/T520) to either alanine or glutamate showed higher activity in the luciferase assay. Considering these mutations resulted in loss of NRD-DBD association, we propose that disruption of the repressive interaction leads to the observed more efficient B-Myb transactivation. We also tested two other mutations at Cdk consensus sites in the NRD, one of which was previously shown to regulate B-Myb by modulating the repressive activity of the NRD (31). However, in our assay, we found that T538 mutation did not change B-Myb activity significantly from that of WT and that S577 mutation resulted in a subtle, albeit significant, reduction of activity.

## Discussion

**Figure 6:**
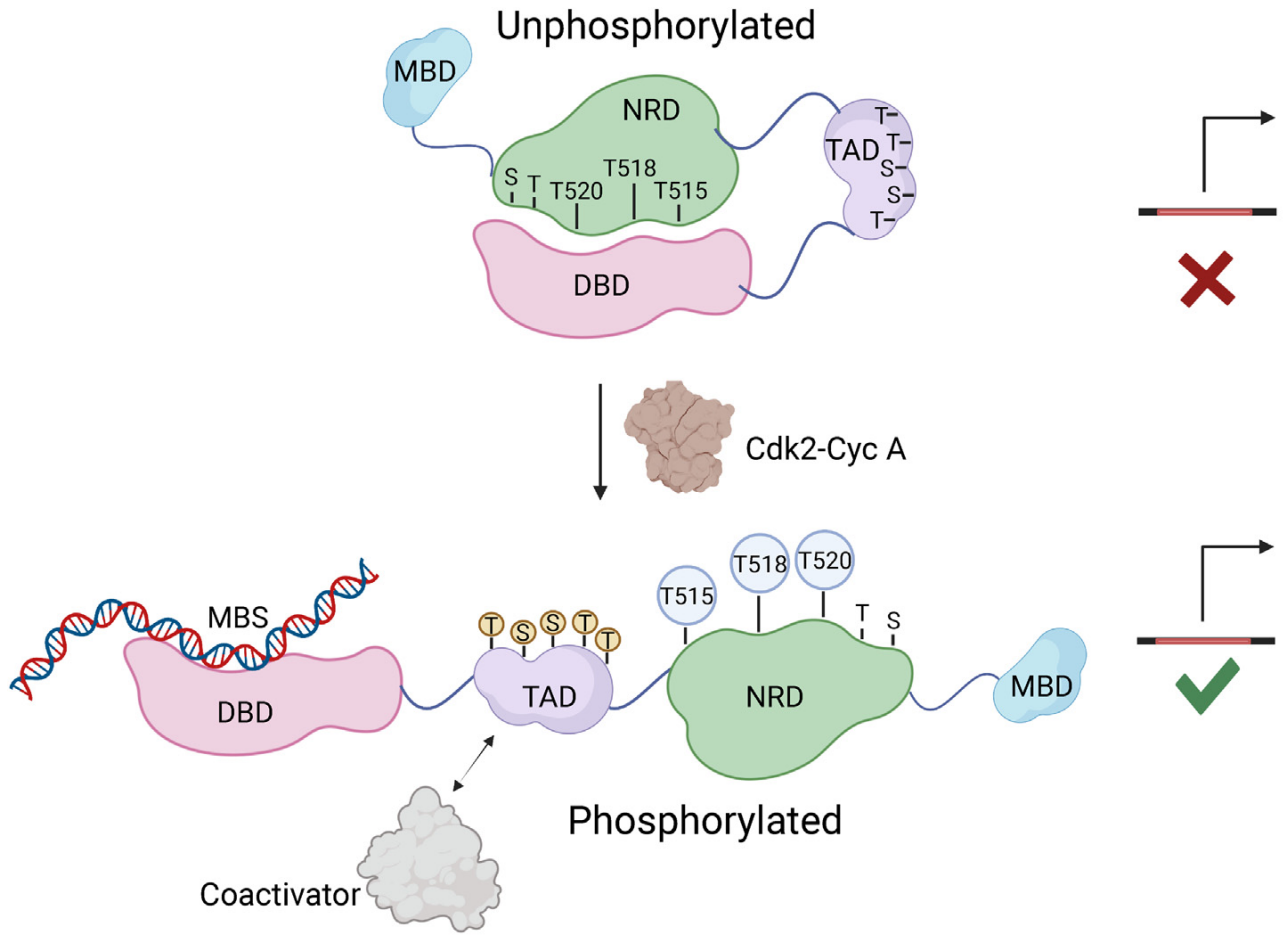
**Structural model for B-Myb autoinhibition and activation upon Cdk2 phosphorylation.** Autoinhibition results from an interdomain association of the NRD and DBD. Cdk2 phosphorylation inhibits this association, releasing the DBD for promoter association. In the canonical model for B-Myb activation, TAD association with coactivators stimulates gene expression. DBD, DNA-binding domain; NRD, negative regulatory domain; TAD, transactivation domain.

Our data show that B-Myb binding to an MBS DNA sequence is inhibited by the intramolecular association between the DBD and the NRD region between 510 and 600 (Fig. 6). This inhibited conformation is regulated by Cdk2-CycA–dependent phosphorylation of T515, T518, and T520, which disrupts the interdomain interaction between the NRD and DBD and permits stronger DNA association. Our mechanistic findings are generally consistent with a number of studies demonstrating, primarily using cell-based reporter assays, that B-Myb transactivation of MBS promoters is autoinhibited by the NRD and increased by cotransfection with CycA (17, 25, 26, 27, 34, 35, 38). Moreover, our biochemical data that Cdk phosphorylation specifically modulates DNA binding offers mechanistic explanation for previous observations that B-Myb phosphorylation and localization to target promoters are coincident (24).

We note several differences between our findings here monitoring the behavior of purified proteins and previous results from cell-based assays. For example, previous studies determined that C-terminal truncations starting from D561 cause the strongest hyperactivity of B-Myb toward a promoter containing Myb-binding sites (17, 27). Cotransfection with Cdk2-CycA further stimulated the activity of the truncations; however, the activity of full-length B-MYB was much further increased by Cdk2-CycA overexpression (27). More recently, it was reported that the B-Myb DBD interacts with a region in the NRD between 560 and 589 and Cdk-mediated phosphorylation of the residue S577 relieves this inhibition (31). In contrast, our NMR and mutagenesis data from biochemical and reporter assays implicate sequences in the NRD that are N-terminal to D561 as those making primary contact with and regulating the DBD, including sequences around the T515, T518, and T520 phosphorylation sites and the amino acid stretch from L541 to L549 (Fig. 3). Our studies did not find S577 to be involved in regulating the NRD-DBD association or S577E to have a positive effect on MBS promoter activity. Rather, we found the more conserved T515/T518/T520 as important phosphorylation sites that regulate MBS-dependent activity. In contrast, other studies using reporter-based cell assays found that point mutations of T518/T518/T520 inhibit MBS transactivation (26, 30, 39); however, it should be noted that other phosphorylation sites were mutated in addition to these sites and may function through independent mechanisms. As an overarching explanation to differences between previous studies and our results, which specifically focus on DNA binding, we speculate that other protein interactions or additional posttranslational modifications also account for the importance of the NRD and its phosphorylation for B-Myb activity and regulation.

Our results demonstrate how intrinsically disordered regions (IDRs) in TFs can regulate TF interactions and how this regulation can be modulated through posttranslational modifications. Other examples of IDRs specifically influencing TF binding to DNA include p53, PU1, ETS1, and TFB2M (40, 41, 42, 43). Like B-Myb, other proteins that control the cell cycle are typically phosphorylated at multiple sites in their IDRs by Cdks (44, 45, 46, 47). Multisite Cdk phosphorylation of TFs like B-Myb and their regulators modulates unique functions through significant changes in structure and interdomain interactions. For example, multisite phosphorylation of the retinoblastoma protein (Rb) promotes ordered structure and induces interdomain interactions that compete with binding to E2F transcription factors (46). In contrast, phosphorylation of the mitotic transcription factor FoxM1 by Cdk2-CycA and Plk1 induces an order to disorder transition (45). FoxM1 phosphorylation switches the protein conformation from an inactive to an active state by inhibiting intramolecular interactions. Our observation that B-Myb inhibition *via* the interdomain NRD-DBD association is released upon Cdk phosphorylation aligns with this common theme of regulation in cell-cycle transcription factors through control of interdomain interactions and structural transitions that promote or reduce structural disorder.

Phosphorylation has been shown to regulate activation of the other Myb family transcription factors. c-Myb gets phosphorylated by several kinases other than Cdk2, including CK2, PKA, and mitogen activated protein kinase (48, 49, 50). There is evidence that the NRDs of c-Myb and A-Myb regulate DNA binding like we have observed here for B-Myb; for example, C-terminal truncations show increased DNA binding and activation of the Myb-dependent reporter promoter *mim-1* (18, 51). The NRD of c-Myb interacts with its DBD though a conserved EVES motif; however, this inhibitory interaction is stimulated by phosphorylation of S581 (the S in EVES) (48, 52). In fact, phosphorylation-dependent rescue of the negative regulation of NRD is not reported for either A-Myb or c-Myb. Therefore, even though Myb proteins have an evolutionarily conserved DBD and recognize the same DNA motif, different phosphorylation patterns and mechanisms may regulate unique tissue-specific functions.

Our study and most previous studies of B-Myb activation have considered B-Myb as a site-specific transcription factor that transactivates from MBS sites, and thus gene expression assays primarily have monitored artificial Myb-responsive reporter genes (17, 35, 36) or type I collagen promoter activity (25). Some studies of B-Myb in the context of activating cell-cycle transcription have led to a model for B-Myb function in which it acts independently of DNA binding to the MBS site. In particular, the MBS site is not essential to activate genes that show cell-cycle–dependent expression and contain the CHR site that is bound by MuvB (53). In contrast, in the context of cell-cycle–dependent gene regulation, the association of B-Myb with MuvB is critical for activation and proper localization of B-Myb to promoters (23, 24, 54). In one remarkable example in flies, expression of the MuvB-binding domain is necessary and sufficient to restore the activity of the PLK1 promoter after loss of B-Myb, suggesting that the DBD is not necessary for B-Myb function in activating some genes (22). It may be then that activation of B-Myb’s ability to bind DNA by Cdk-mediated phosphorylation is a particular mechanism for MBS promoters, while activation of CHR promoters may entail other additional mechanisms. Nevertheless, a slow migrating phosphorylated form of B-Myb is abundantly found in S-phase of the cell cycle, which coincides with B-Myb expression and localization to cell-cycle dependent promoters (29, 35, 55). Thus, it is possible that phosphorylation might be enhancing other protein–protein interactions, like B-Myb-FoxM1, that are specifically necessary for cell-cycle–dependent gene activation (24). Further studies on B-Myb will clarify how it activates cell-cycle–dependent genes at the CHR promoters.

## Experimental procedures

### Recombinant protein expression and purification

The human B-Myb full-length protein and B-Myb^34–600^ constructs were expressed in Sf9 cells with a cleavable N-terminal Strep tag using the FastBac expression system. Cells were harvested and lysed in a buffer containing 300 mM NaCl, 50 mM Tris, 1 mM DTT, 10% Glycerol v/v, Sigma Protease Inhibitor (P8340), and 1 mM PMSF (pH 8.0). Protein was purified with StrepTactin Sepharose High Performance resin (Cytiva) equilibrated in lysis buffer. The lysed cells were clarified by centrifugation at 19,000 rpm for 45 min at 4 °C. The cleared lysate was incubated with resin for 1 h at 4 °C, and the resin was washed with a buffer containing 300 mM NaCl, 50 mM Tris, 1 mM DTT, and 10% glycerol v/v (pH 8.0). The protein was then eluted in 300 mM NaCl, 50 mM Tris, 5 mM desthiobiotin, 10% glycerol v/v, and 1 mM DTT (pH 8.0). Protein was dialyzed into storage buffer (200 mM NaCl, 50 mM Tris, 1 mM BME, and 10% glycerol v/v (pH 8.0)) and stored at −80 °C.

The human B-Myb–truncated constructs (DBD, TAD, NRD, B-Myb^34–370^) were expressed in *Escherichia coli* from an engineered pGEX plasmid with an N-terminal GST tag and a TEV protease cleavage site. Proteins were expressed overnight by inducing with 1 mM IPTG at 19 °C. All proteins were lysed in a buffer containing 200 mM NaCl, 40 mm Tris, 5 mM DTT, and 1 mM PMSF (pH 8.0). The lysed cells were clarified by centrifugation at 19,000 rpm for 45 min at 4 °C. Protein lysates were allowed to bind to equilibrated Glutathione Sepharose resin (Cytiva) for 30 min and washed to remove unspecific proteins. The proteins were eluted with a buffer containing 200 mM NaCl, 40 mM Tris, 5 mM DTT, and 10 mM reduced L-Glutathione (pH 8.0). Eluted proteins were further purified using Q-sepharose and cleaved with TEV protease at 4 °C overnight. Proteins were then passed through Glutathione Sepharose resin to remove the free GST and concentrated to run through Superdex-75 (GE Healthcare) into 200 mM NaCl, 25 mM Tris, and 1 mM DTT (pH 8.0). Cdk2-CycA and Plk1 kinase domains were expressed and purified as previously described (45).

To generate phosphorylated protein reagents, kinase reactions were performed similar to as previously described (45). B-Myb protein constructs following final purification were incubated with 10 mM ATP, 50 mM MgCl_2_, and 20% by mass of either Cdk2-CycA, Plk1 kinase domain, or both Plk1 and Cdk2-CycA, overnight at 4 °C. The kinase reaction was concentrated and run over Superdex-75 (GE Healthcare) to remove kinases and ATP, and phosphorylation of the proteins was confirmed by electrospray mass spectrometry using a Sciex X500B QTOF system.

### FP assay

Dissociation constants for direct binding between DBD and MBS DNA sequence were determined by titrating increasing amounts of DBD into 20 nM of TAMRA dye-labeled MBS DNA probe. The duplex DNA probe was synthesized by Integrated DNA Technologies and had the following sequence: 5′-GCATTATAACGGTCTTTTAGCGCCTGG/36-TAMSp/-3′. For DBD + NRD assays, DBD and NRD were incubated for 30 min on ice before titrating the labeled MBS probe in a buffer containing 150 mM NaCl, 25 mM Tris, 1 mM DTT, and 0.1% Tween20 (pH 8). FP measurements were acquired on a PerkinElmer EnVision 2103 Multilabel plate reader with excitation at 559 nm and emission at 580 nm. The dissociation constants (K_D_) were calculated by fitting millipolarization (mP) values of three technical replicates against concentration using a one site–binding model in GraphPad Prism 8.

### Isothermal titration calorimetry

Dissociation constants (K_D_) for DBD and NRD interactions were measured using ITC with a MicroCal VP-ITC system. All proteins were concentrated as needed and dialyzed into a buffer containing 150 mM NaCl, 20 mM Tris, and 1 mM BME (pH 8). DBD (500 μM) was titrated into NRD (50 μM) at 19 °C. The dissociation constant of NRD mutants and phosphorylated NRDs were determined similarly. K_D_s are the average fits from three technical replicates analyzed using the Origin ITC software package with the SD reported as error. All the fit stoichiometry (n) values were between 0.6 and 1.

### NMR spectroscopy

The HSQC and CON spectra for DBD and NRD interaction studies in Figure 3 were collected at 25 °C on a Bruker Avance III HD 800-MHz spectrometer equipped with a cryogenically cooled probe. The sample contained ^13^C-^15^N-labeled NRD^510–600^ at 300 μM in a buffer containing 20 mM sodium phosphate pH 8.0, 100 mM NaCl, 1 mM DTT, and 5% (v/v) D_2_O. The backbone assignment of the NRD was accomplished using standard NH-edited triple-resonance experiments [HNCO, HNCACB, CBCA(CO)NH, C(CO)NH] supplied by Varian/Agilent (33). The NH-edited experiments were collected on a Varian/Agilent INOVA 600 MHz NMR equipped with a cryogenically cooled probe. The experiments for backbone assignments were collected at pH 6.0 (otherwise same buffer) due to favorable chemical exchange, and assignments were transferred to pH 8.0 CON spectra through pH titrating. The 1D ^1^H spectra were acquired using the Avance III HD 800-MHz NMR system. Samples contained 300 μM NRD in a buffer containing 50 mM sodium phosphate pH 6.0, 100 mM NaCl, 1 mM DTT, and 10% (v/v) D_2_O. All spectra were processed using NMRPipe and analyzed and assigned using Sparky (56, 57).

### Cell culture, luciferase assays, and Western blot

HCT116 colon carcinoma cells were grown in Dulbecco's modified Eagle's medium (Gibco, high glucose, GlutaMAX Supplement, pyruvate) supplemented with 10% fetal bovine serum (Corning, Regular Fetal Bovine Serum) and penicillin/streptomycin (Gibco). Cells were maintained at 37 °C and 5% CO_2_.

The 3xMBS luciferase reporter construct was created by inserting a double-stranded oligonucleotide containing three copies of a high affinity B-Myb–binding site (TAACGGTG) (1, 2, 3, 4) upstream of the herpes simplex thymidine kinase minimal promoter (5-TTA**TAACGGTC**TTAA**TAACGGTC**TTAA**TAACGGTC**TTTTAGC*TTCGCATATTAAGGTGACGCGTGTGGCCTCGAACACCGAGCGACCCTGCAGCGACCCGCTTAA*-3; MBSs in bold, minimal TK promoter in italics) into the KpnI and NcoI sites of the pGL4.10[luc2] vector (Promega). The ORF of human MYBL2/B-Myb isoform 1 (NM_002466.4) was cloned into pcDNA3.1+ (ThermoFisher Scientific) and fused with an N-terminal Flag tag. Point mutations were introduced following the QuikChange site-directed mutagenesis protocol, and the DBD (amino acids 12-182) was deleted following the NEB Q5 protocol.

Stimulation of the 3xMBS promoter activity was analyzed by luciferase reporter assays with extracts of transfected HCT116 cells. Thirty thousand cells per 48 wells were plated and transfected with 1 μl PEI (Polysciences, PEI 25K), 75 ng of promoter reporter plasmids (3xMBS-pGL4.10 or pGl4.10 empty vector), 100 ng of pcDNA3.1 plasmids expressing Flag-B-Myb (WT or mutants), and 25 ng renilla luciferase plasmid (pGL4.70). Cells were lysed 48 h after transfection, and luciferase activity was measured with the Dual-Luciferase Reporter Assay System (Promega) following the manufacturer's recommendations on an EnVision 2105 plate reader (PerkinElmer). Relative promoter activities of the 3xMBS-pGL4.10 reporter after expression of WT or mutant B-Myb were calculated by normalizing to renilla luciferase activity and to the activity of the pGL4.10 empty vector cotransfected with the respective B-MYB constructs.

Expression levels of WT and mutant B-Myb were analyzed by loading 10 μg of the remaining luciferase assay lysates onto a 10% SDS gel followed by Western blotting. Flag-B-Myb was detected with the Anti-OctA-Probe antibody (Santa Cruz Biotech, sc-166355 HRP, dilution 1:2000), and β-actin was probed with the Direct-Blot HRP anti-β-actin antibody (BioLegend, clone W16197A, Cat. # 664804, dilution 1:10,000).

## Data availability

NMR chemical shift data for B-Myb NRD^510–600^ have been deposited to the Biological Magnetic Resonance Data Bank under accession code 51427.

## Supporting information

This article contains supporting information.

## Conflicts of interest

The authors declare that they have no conflicts of interest with the contents of this article.
