# Supplementary material for: Cyclin-dependent kinase-mediated phosphorylation and the negative regulatory domain of transcription factor B-Myb modulate its DNA binding
Source: J Biol Chem. 2022 Aug 1;298(9):102319. doi: 10.1016/j.jbc.2022.102319 (PMC9478404; doi:10.1016/j.jbc.2022.102319)
Supplement: Supplemental Figures S1–S5 [file mmc1.pdf]

Supplementary Information

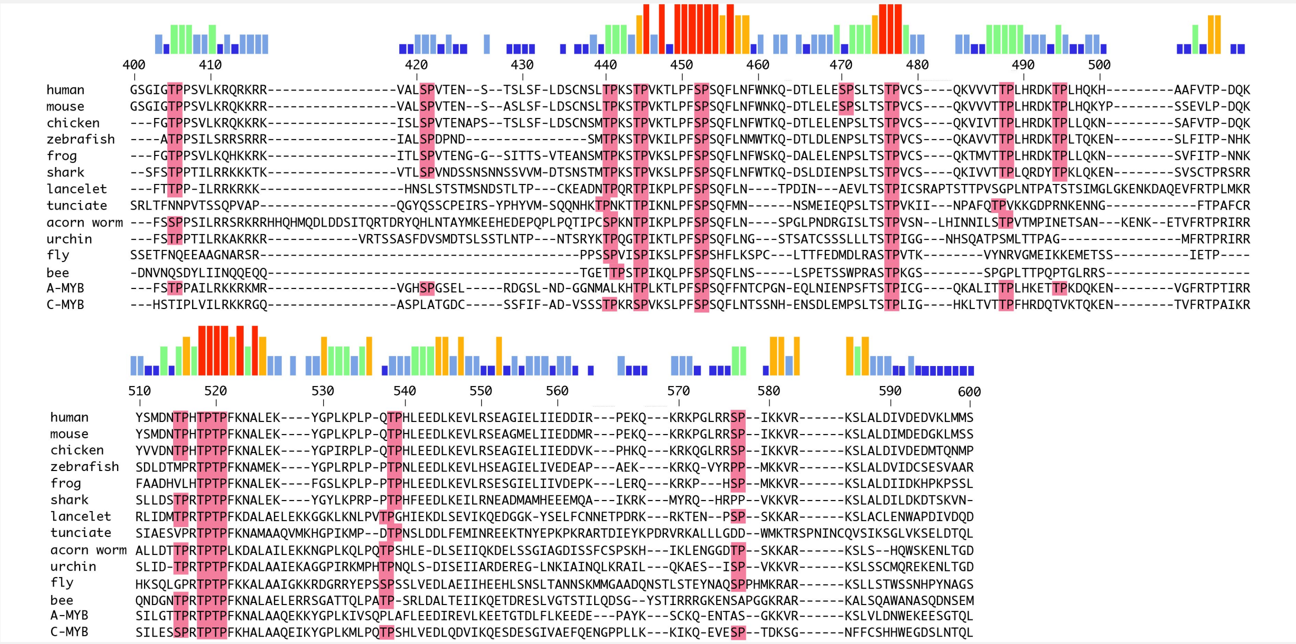

Figure S1. Sequence alignment of the B-Myb NRD. Sequences for B-Myb orthologs are shown along with the sequences of human A-Myb and c-Myb. Minimal Cdk sites consensus sites (S/TP) are highlighted in pink. The B-Myb proteins sequences used are from human (Homo sapiens), mouse (Mus musculus), chicken (Gallus gallus), zebrafish (Danio rerio), frog (Xenopus laevis), shark (Callorhinchus milii), lancelet (B. floridae), tunicate (Ciona intestinalis), acorn worm (Saccoglossus kowalevskii), urchin (Strongylocentrotus purpuratus), fly (Drosophila melanogaster), and bee (Apis mellifera).

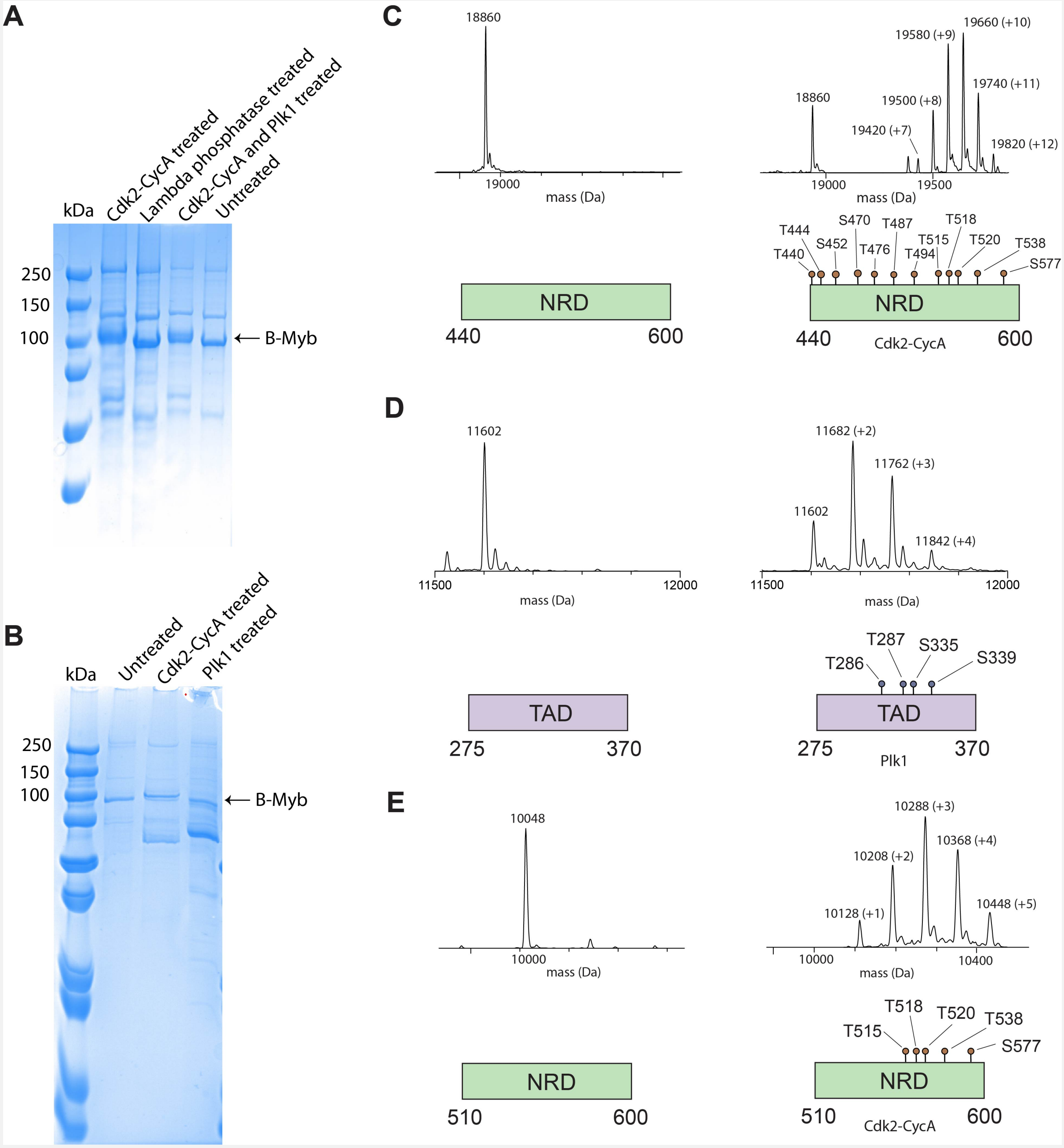

Figure S2. Confirmation of recombinant protein phosphorylation. (A-B) PhosTag gels following purification of full-length B-Myb expressed in Sf9 cells and treatment with the indicated enzyme(s). The B-Myb band appears with similar mobility in the phosphatase treated and untreated samples, suggesting that untreated purified protein is not extensively phosphorylated. Phosphorylation with Cdk2-CycA produces a noticeable shift, whereas the shift with Plk1 treatment is not detectable when using Plk1 alone or together with Cdk2-CycA. However, we are confident in the activity of our Plk1 enzyme preparation considering our mass spectrometry results detecting TAD phosphorylation in panel D. (C) B-Myb NRD$^{440-600}$ unphosphorylated (left) and phosphorylated (right) by Cdk2-Cyc A detected by mass spectrometry. In this construct there are 3 strong Cdk consensus sites ({S/T}Px{K/R}) at T444, T520 and S577 and 9 other weak consensus sites ({S/T}P). Peaks are labeled with the final mass and the number of phosphoryl groups added in parenthesis (+80 Da). (D) B-Myb TAD unphosphorylated (left) and phosphorylated (right) by Plk1. This construct contains 4 Plk1 consensus sites ({D/N/E/Q}xSΦ) as depicted in the schematic diagram. (E) B-Myb NRD$^{510-600}$ unphosphorylated (left) and phosphorylated (right) by Cdk2-Cyc A detected by mass spectrometry.

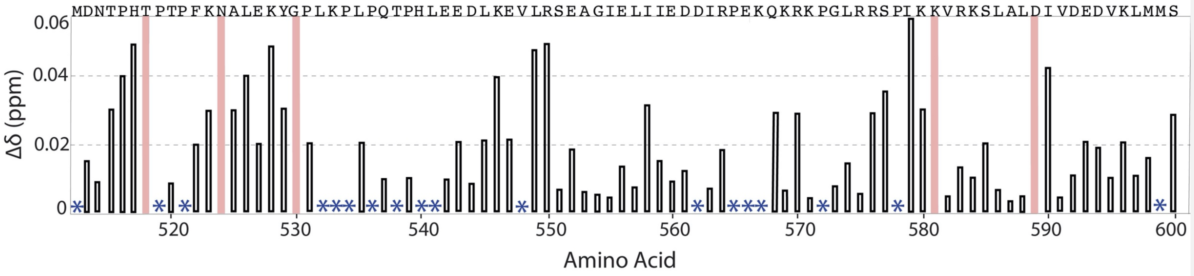

Figure S3. Chemical shift perturbation plot of NRD-DBD association. Chemical shift change ($\Delta\delta$) of the corresponding peak for each amino acid in the ^{13}C-^{15}N CON spectrum. The change compares the labeled NRD alone to the labeled NRD plus unlabeled DBD. $\Delta\delta$ is calculated using $\Delta\delta = \sqrt{((0.3\Delta N)^2 + \Delta C^2)}$, where ΔN and ΔC are the shift differences in each dimension and the 0.3 normalizes to the different nuclear gyromagnetic ratios. Overall the perturbations are modest compared to the observed loss in peak intensities shown in Fig. 3D, although regions near the T515/T518/T520 phosphorylation sites and conserved L541/L545/L549 residues show relatively larger changes that is consistent with the peak intensity analysis. Asterisks mark amino acids that could not be assigned. Red bars mark residues corresponding to peaks that completely disappear in the spectrum with DBD.

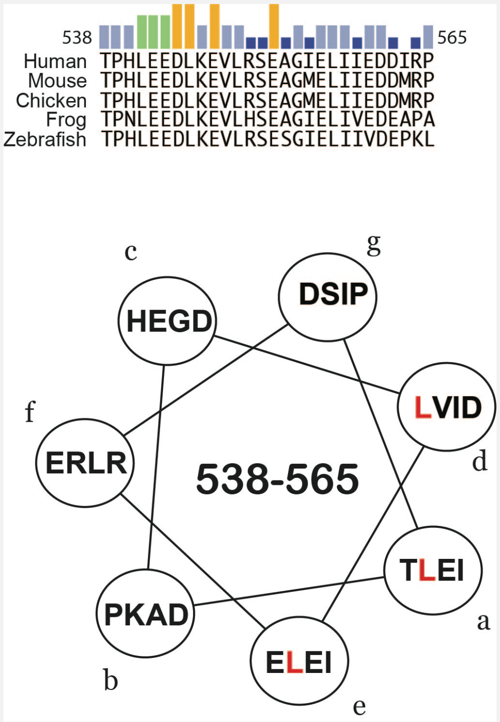

Figure S4. Rational for probing the L541/L545/L549 NRD mutant. (Top) Sequences for five B-Myb orthologs. The bars on top of the sequence reflect conservation from the larger sequence alignment shown in Fig. S1. (Bottom) Helical wheel projection beginning with T538 in the "a" position. The projection suggests that this sequence has the potential to form an amphipathic helix with the three leucines (colored in red) positioned along one face.

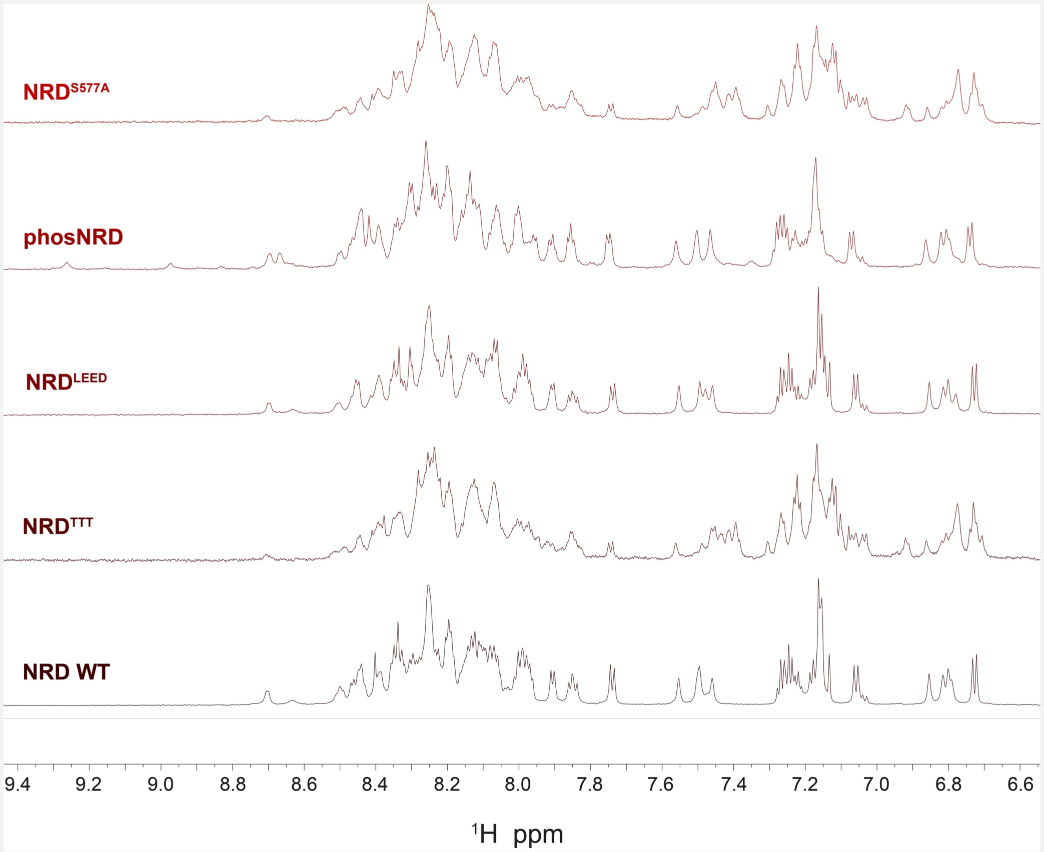

Figure S5. ^{1}H-NMR spectra of B-Myb NRD domains. 1D ^{1}H-NMR spectra were acquired for the indicated NRD construct at 300 μM: unphosphorylated WT (NRD WT), phosphorylated WT (phosNRD), S577A phosphorylation site mutant (NRD^{S577A}), L541A/E542A/E543A/D544A mutant (NRD^{LEED}), and T515A/T518A/T520A phosphorylation site mutant (NRD^{TTT}). The spectra show several chemical shift changes for select peaks from site specific phosphorylation or mutagenesis; for example, the downfield shifts of several amide protons in the phosNRD spectrum from sidechain phosphorylation are observable. However, there are no gross overall differences in the spectra, and the limited but similar chemical shift dispersion is consistent will all the constructs adopting similar distributions of disordered conformations.
